# Epidemiology and management of infections in critically ill neonates: findings from a cohort study in a Brazilian neonatal intensive care unit

**DOI:** 10.1099/jmm.0.001968

**Published:** 2025-03-25

**Authors:** Isadora Caixeta da Silveira Ferreira, Ralciane de Paula Menezes, Mallu Santos Mendonça Lopes, Lúcio Borges de Araújo, Daniela Marques de Lima Mota Ferreira, Denise Von Dolinger de Brito Röder

**Affiliations:** 1 Postgraduate Program in Health Sciences, Faculty of Medicine, Federal University of Uberlândia, Uberlândia, Minas Gerais, Brazil; 2 Department of Microbiology, Institute of Biomedical Sciences, Federal University of Uberlândia, Uberlândia, Minas Gerais, Brazil; 3 Undergraduate Course in Biomedicine, Institute of Biomedical Sciences, Federal University of Uberlândia, Uberlândia, Minas Gerais, Brazil; 4 Faculty of Mathematics, Federal University of Uberlândia, Uberlândia, Minas Gerais, Brazil; 5 Neonatal Intensive Care Unit, Federal University of Uberlândia, Uberlândia, Minas Gerais, Brazil

**Keywords:** birth weight, infant health, infection control, perinatal death, premature birth

## Abstract

**Introduction.** Healthcare-associated infections (HAIs) are the leading cause of preventable death in neonatal intensive care units (NICUs), particularly among very low birth weight (VLBW) infants.

**Hypothesis/Gap Statement**: VLBW neonates are at higher risk of HAIs, particularly those caused by Gram-negative bacteria (GNB) and fungi, which can negatively impact their survival and prolong hospitalization.

**Aim.** To determine the risk factors, aetiology, antimicrobial susceptibility and clinical outcomes of HAIs in VLBW neonates in a Brazilian NICU.

**Methodology.** This retrospective cohort study analysed the medical records of VLBW newborns admitted to the NICU from January 2015 to December 2022.

**Results.** Among VLBW neonates, 269/670 (40.1%) developed HAIs and 203/670 (30.3%) developed sepsis. The incidence of HAIs (54.5% vs. 36.2%) and sepsis (49.7% vs. 25%) was higher in neonates weighing less than 750 g. The median birth weight of infected newborns was 960 g, and the median age of infection onset was 16 days. There were 292/456 (64%) infections caused by Gram-positive bacteria, 138/456 (30.3%) by GNB and 26/456 (5.7%) by fungi. Of the isolates, 277/456 (60.7%) were multidrug-resistant. Newborns weighing less than 750 g or infected with GNB and/or fungi had lower survival rates. Previous use of antifungals was the main predictor of infection (*P*<0.01; odds ratio=4.21). Infections prolonged hospital stay from 25 to 42 days. The mortality rate was 17.6%, with a case lethality rate of 16.4%; 75% of deaths in the infected group were due to sepsis.

**Conclusion.** The high incidence of infection emphasizes the need for infection control and antimicrobial management. Low birth weight is associated with increased risk of infection and decreased survival. The increase in GNB and fungal infections requires prevention and treatment strategies to reduce neonatal morbidity and mortality.

## Introduction

Annually, ~2.4 million newborn deaths occur worldwide [1], with one-third of these deaths attributed to healthcare-associated infections (HAIs), which represent the leading cause of death in neonatal intensive care units (NICUs) [2]. These infections can progress to sepsis, affecting 3 million newborns annually [3].

Birth weight is a significant predictor of newborn sepsis, particularly among very low birth weight (VLBW) neonates due to the interplay of factors associated with prematurity, an immature immune system, feeding difficulties and an elevated risk of hypoglycaemia [4]. These factors collectively contributed to an increased incidence of HAIs and early neonatal mortality, with far-reaching socioeconomic consequences [5].

A review of the literature reveals a correlation between increased hospitalization periods and elevated mortality rates among VLBW newborns: in South Africa, the average length of stay is 44 vs. 38 days, with the mortality rate of 28.1% vs. 5.3% [6]; in the USA, 16% vs. 7% mortality [7]; in Brazil, 68 vs. 44 days and 26.6% vs. 9.4% mortality [8].

Given that VLBW affects 15%–20% of newborns globally and is associated with increased morbidity and mortality [9], this study aims to provide data for the management of HAIs among these patients. It seeks to identify risk factors for HAIs, the aetiological agents involved, antimicrobial susceptibility and the clinical outcomes of infected newborns.

## Methods

### Definitions

The newborns were categorized according to their respective birth weights, as follows [10]:

VLBW: 1,000–1,499 g.

Extremely low birth weight: <1,000 g.

The newborns were classified according to gestational age as follows [10]:

Preterm: <37 weeks.

Moderate or late preterm: 32–36 weeks.

Very preterm: 28–31 weeks.

Extremely preterm: <28 weeks.

Regarding antimicrobial resistance, the isolates were classified as follows:

Carbapenem-resistant: resistance to at least one of the tested antimicrobials in this category [11];

Extended-spectrum beta-lactamase producers: production of enzymes that confer resistance to a range of beta-lactams, including penicillins, third-generation cephalosporins and monobactams such as aztreonam [12];

Multidrug-resistant (MDR): resistance to at least one antimicrobial in three or more categories, or *Staphylococcus* spp. resistant to oxacillin (ORS) [13];

Extensively drug-resistant (XDR): resistance to at least one antimicrobial in all tested categories, except two or fewer [13].

The incidence of HAIs was evaluated using the incidence rate [14] and incidence per 1,000 patient days [15]. The mortality rate was defined as the proportion of deaths among all participants, and the case lethality rate was defined as the proportion of deaths among participants who developed HAIs [16].

### Type and location of study

This is a retrospective observational cohort study, utilizing data from the medical records of newborns admitted to the NICU at the Hospital de Clínicas da Universidade Federal de Uberlândia (HC-UFU) from 1 January 2015 to 31 December 2022. This public hospital, which has 520 beds, provides tertiary care, and its NICU, which is recognized as a regional reference for neonatal care, has 20 beds. The study was approved by the Ethics Committee for Human Research at UFU (no. 6647697/2024).

### Population and study design

This study included VLBW newborns who had been alive for a minimum of 72 h and were monitored daily through the National Healthcare Safety Network form [17]. The data encompassed biological sex, birth weight, gestational age, type of delivery, premature rupture of membranes (PROMs), congenital malformations, length of hospital stay, use of antimicrobials/antifungals, invasive devices, surgical procedures and clinical outcomes. Regarding HAIs, the analysis covered the previous and subsequent hospitalization periods until the outcome, isolated species, antimicrobial susceptibility and affected site.

To analyse the risk factors for infection, the participants were grouped into two categories: non-infected (VLBW newborns without infection) and infected (VLBW newborns with microbiologically confirmed infection). Infected newborns were further subdivided into those who were discharged from the NICU and those who died to identify predictors of mortality. For other analyses, infected VLBW newborns were classified into group 1 (birth weight less than 750 g) and group 2 (birth weight between 750 and 1,499 g).

### Microbiological profile and antimicrobial resistance

The presence of bloodstream infections was confirmed through the utilization of the BACT/Alert^®^ system (bioMérieux, EUA). In the case of other infections, samples were cultured on blood agar plates to isolate the causative agent. Identification and antimicrobial susceptibility profiles were determined using the VITEK^®^ 2 system (bioMérieux, France), in accordance with the guidelines set forth by the Brazilian Committee on Antimicrobial Susceptibility Testing. Clinical sample collection was conducted in accordance with local medical protocols.

### Data analysis

Quantitative variables were described using median and interquartile range (IQR), while qualitative variables were expressed as frequency and percentage. The potential risk factors for infection and predictors of mortality were assessed through univariate and multivariate logistic regression, with a significance level of 5% (*P*<0.05). The analyses were conducted using IBM SPSS for Windows^®^ v.20 (IBM Corp., Armonk, USA). The Kaplan–Meier method was employed to estimate the probability of survival of infected newborns.

## Results

### Characterization of the population

Among the 670 VLBW newborns included in this study, 269 (40.1%) developed HAIs and 203 (30.3%) developed sepsis. The median age at the onset of infection was 16 days (IQR: 10–27), ranging from 18 days (IQR: 8–32) in group 1 to 16 days (IQR: 11–26) in group 2. Among the infected, 52.8% (142/269) were male, 29% (78/269) had a birth weight of less than 750 g, and 47.5% (125/263) were extremely preterm. The majority of the subjects (71.8%; 191/266) were delivered by caesarean section, and 13.9% (32/231) had PROM ≥48 h before delivery. Additionally, 24.2% (65/269) exhibited some congenital malformation, as detailed in Table 1.

**Table 1.** Risk factors associated with infection in neonates with VLBW (<1,500 g)

Variable	Non-infected		Infected		Univariate model		Multivariate model	
	*N*	%	*N*	%	OR(95% CI)	*P*	OR(95% CI)	*P*
**Neonates**	401	59.9	269	40.1	–	–	–	–
**Biological sex**								
Male	204	50.9	142	52.8	–	–	–	–
Female	197	49.1	127	47.2	0.93 (0.68–1.26)	0.63	–	–
**Birth weight** (g)								
<750	65	16.2	78	29.0	2.11 (1.45–3.07)	0.01*	–	–
750–999	93	23.2	72	26.8	1.21 (0.85–1.73)	0.29	–	–
1,000–1,499	243	60.6	119	44.2	0.52 (0.38–0.71)	0.01*	–	–
Median IQR	1,095 (870–1,300)		960 (696–1,190)		–	–	–	–
**Gestational age** (weeks)								
<28	123	32.0	125	47.5	1.92 (1.39–2.66)	0.01*	–	–
28–31	192	50.0	117	44.5	0.8 (0.58–1.1)	0.17	–	–
32–36	66	17.2	21	8.0	0.42 (0.25–0.7)	0.01*	–	–
≥37	3	0.8	0	0.0	1 (–)	0.98	–	–
**Mode of delivery**								
Caesarean	290	74.4	191	71.8	0.88 (0.62–1.25)	0.47	–	–
Vaginal	100	25.6	75	28.2	–	–	–	–
**PROM** (h)								
<48	300	88.5	199	86.1	–	–	–	–
≥48	39	11.5	32	13.9	1.24 (0.75–2.04)	0.41	–	–
**Congenital malformation**	59	14.7	65	24.2	1.85 (1.25–2.74)	0.01*	–	–
**Surgical procedure**	33	8.2	26	9.7	1.19 (0.7–2.05)	0.52	–	–
**Referred from another hospital**	19	4.7	15	5.6	1.19 (0.59–2.38)	0.63	–	–
**Stay in NICU,** median IQR (days)	25 (13–47)		42 (26–69)		1.02 (1.02–1.03)	0.01*	1.01(1.01–1.02)	0.01*
**Empirical prescription of antimicrobials**	243	60.6	166	61.7	1.05 (0.76–1.44)	0.77	–	–
Median IQR (days)	7 (4–11.5)		7 (5–10)		0.98 (0.95–1)	0.10	0.85(0.81–0.9)	0.01*
Penicillins	236	58.9	156	58.0	0.97 (0.71–1.32)	0.82	–	–
Median IQR (days)	7 (4–10.3)		7 (4–9)		0.97 (0.93–1)	0.09	–	–
Aminoglycosides	212	52.9	142	52.8	1 (0.73–1.36)	0.98	–	–
Median IQR (days)	6 (4–8)		5.5 (4–8)		0.95 (0.9–1.01)	0.12	–	–
Third-generation cephalosporins	47	11.7	30	11.2	0.95 (0.58–1.54)	0.82	–	–
Median IQR (days)	5 (3.5–8)		6 (4–8)		0.99 (0.9–1.1)	0.90	–	–
Fourth-generation cephalosporins	28	7.0	24	8.9	1.31 (0.74–2.3)	0.36	–	–
Median IQR (days)	7.5 (3.8–12.3)		6 (2–8)		0.92 (0.83–1.03)	0.15	–	–
Glycopeptides	29	7.2	16	6.0	0.82 (0.44–1.54)	0.54	–	–
Median IQR (days)s	8 (4–11)		6 (4–10)		0.99 (0.9–1.08)	0.78	–	–
Nitroimidazoles	9	2.2	7	2.6	1.16 (0.43–3.16)	0.77	–	–
Median IQR (days)	7 (5–10)		5 (2–8.5)		0.82 (0.61–1.11)	0.20	–	–
**Empirical prescription of antimicrobials**	23	5.7	51	19	3.84 (2.29–6.47)	0.01*	4.21(1.85–9.58)	0.01*
Median IQR (days)	8 (6–9)		8 (6.5–11)		1.08 (0.97–1.2)	0.18	–	–
**Use of PN**	379	94.5	262	97.4	2.17 (0.91–5.16)	0.08	–	–
Median IQR (days)	12 (9–15)		16 (12–23.8)		1.09 (1.06–1.11)	0.01*	1.06(1.02–1.1)	0.01*
**Use of PICC**	330	82.3	249	92.6	2.68 (1.59–4.52)	0.01*	–	–
Median IQR (days)	13 (9–18)		19 (13–31)		1.06 (1.04–1.08)	0.01*	1.04(1.01–1.08)	0.01*
**Use of UVC**	307	76.6	219	81.4	1.34 (0.91–1.97)	0.13	–	–
Median IQR (days)	4 (2–6)		5 (3–7)		1.06 (1.01–1.13)	0.03*	–	–
**Use of intubation**	237	59.1	205	76.2	2.22 (1.57–3.13)	0.01*	–	–
Median IQR (days)	5 (2–10)		12 (4–30)		1.04 (1.02–1.05)	0.01*	–	–
**Use of drain**	17	4.2	21	7.8	1.91 (0.99–3.7)	0.05	–	–
Median IQR (days)	6 (4–8)		8 (5–11)		1.16 (0.97–1.38)	0.10	–	–
**Use of bladder catheter**	7	1.7	14	5.2	3.09 (1.23–7.76)	0.02*	–	–
Median IQR (days)	2 (1.5–10)		3.5 (2–7)		1 (0.86–1.17)	0.96	–	–

Among the non-infected neonates, data were available on gestational age for 384, on type of delivery for 390 and on PROM for 339, whereas among the infected neonates, data were available on gestational age for 263, on type of delivery for 266 and on PROM for 231. All information before infection refers to the first episode.

* *P*<0.05.

CIs, confidential intervals; OR, odds ratio; PICC, peripherally inserted central catheter; PN, parenteral nutrition; UVC, umbilical venous catheter

A significant number of infected newborns discharged from the NICU received antimicrobial therapy (80%; 180/225), with a median duration of 13 days (IQR: 9–20). Among the infected newborns who died, 77.3% (34/44) were treated with antimicrobials, with a median duration of 8.5 days (IQR: 5–15). The therapeutic use of antifungals varied between the two groups, with a prevalence of 7.1% (16/225) among neonates discharged from the NICU and 15.9% (7/44) among those who died (Table 2).

**Table 2.** Predictors of fatality in neonates with VLBW (<1,500 g)

Variable	Infected discharge		Infected dead		Univariate model		Multivariate model	
	*N*	%	*N*	%	OR (95% CI)	*P*	OR (95% CI)	*P*
**Neonates**	225	83.6	44	16.4	–	–	–	–
**Biological sex**								
Male	122	54.2	20	45.5	–	–	–	–
Female	103	45.8	24	54.5	1.42 (0.74–2.72)	0.29	–	–
**Birth weight** (g)								
<750	52	23.1	26	59.1	4.81 (2.44–9.45)	0.01*	–	–
750–1,499	173	76.9	18	40.9	0.21 (0.11–0.41)	0.01*	–	–
Median IQR	980 (770–1,210)		668 (611.5–1,001.3)		1 (1–1)	0.01*	–	–
**Gestational age** (weeks)								
<28	95	43.0	30	71.4	3.32 (1.61–6.82)	0.01*	–	–
28–31	108	48.9	9	21.4	0.29 (0.13–0.62)	0.01*	0.15 (0.03–0.8)	0.03*
32–36	18	8.1	3	7.1	0.87 (0.24–3.09)	0.83	–	–
**Mode of delivery**								
Caesarean	163	73.4	28	36.4	0.63 (0.32–1.25)	0.19	–	–
Vaginal	59	26.6	16	63.6	–	–	–	–
**PROM** (h)								
<48	171	86.8	28	82.4	–	–	–	–
≥48	26	13.2	6	17.6	1.41 (0.53–3.73)	0.49	–	–
**Congenital malformation**	57	25.3	8	18.2	0.66 (0.29–1.49)	0.31	–	–
**Surgical procedure**	20	8.9	6	13.6	1.62 (0.61–4.29)	0.33	–	–
**Referred from another hospital**	11	4.9	4	9.1	1.95 (0.59–6.42)	0.27	–	–
**Stay in NICU**, median IQR (days)	46 (31–75)		19 (10.8–26.8)		0.95 (0.93–0.97)	0.01*	0.95 (0.91–0.98)	0.01*
**Antimicrobial treatment**	180	80.0	34	77.3	0.85 (0.39–1.85)	0.68	–	–
Median IQR (days)	13 (9–20)		8.5 (5–15)		0.98 (0.94–1.01)	0.19	–	–
Penicillins	141	62.7	30	68.2	1.28 (0.64–2.54)	0.49	–	–
Median IQR (days)	5 (3–11)		7 (2.3–9)		1 (0.95–1.05)	0.97	–	–
Aminoglycosides	131	58.2	22	50.0	0.72 (0.38–1.37)	0.32	–	–
Median IQR (days)	5 (3–9)		3 (2–5)		1 (0.94–1.07)	0.91	–	–
Glycopeptides	108	48.0	14	31.8	0.51 (0.25–1)	0.05*	–	–
Median IQR (days)	9.5 (8–13.3)		10 (6.5–18.3)		1.04 (0.97–1.1)	0.26	1.14 (1.02–1.27)	0.02*
Third-generation cephalosporins	38	16.9	8	18.2	1.09 (0.47–2.54)	0.84	–	–
Median IQR (days)	8.5 (5.3–12)		4.5 (2–6.3)		0.76 (0.59–0.97)	0.03*	–	–
fourth-generation cephalosporins	42	18.7	15	34.1	2.25 (1.11–4.57)	0.02*	5.89 (1.44–24.13)	0.01*
Median IQR (days)	7 (4–12.8)		6 (3–7.5)		0.97 (0.88–1.07)	0.57	–	–
Nitroimidazoles	3	1.3	3	6.8	5.41 (1.06–27.76)	0.04*	–	–
Median IQR (days)	3 (2.5–3.5)		4 (3–5)		1.79 (0.46–7.02)	0.40	–	–
Carbapenems	19	8.4	2	4.5	0.52 (0.12–2.3)	0.39	–	–
Median IQR (days)	14 (6.5–16)		12.5 (11.8–13.3)		1 (0.82–1.22)	0.99	–	–
**Antifungal treatment**	16	7.1	7	15.9	2.47 (0.95–6.42)	0.06	–	–
Median IQR (days)	16.5 (14.8–23)		15 (8–19)		0.93 (0.82–1.06)	0.29	–	–
**Use of intubation**	164	72.9	41	93.2	5.08 (1.52–17.02)	0.01*	–	–
Median IQR (days)	10.5 (3–37.3)		15 (8–23)		1 (0.98–1.01)	0.62	–	–
**Use of PN**	222	98.7	40	90.9	0.14 (0.03–0.63)	0.01*	–	–
Median IQR (days)	16 (12–24)		13.5 (7–19.3)		0.97 (0.93–1)	0.07	–	–
**Use of UVC**	183	81.3	36	81.8	1.03 (0.45–2.38)	0.94	–	–
Median IQR (days)	5 (2–7)		5 (3.8–7)		1.03 (0.92–1.15)	0.60	–	–
**Use of PICC**	214	95.1	35	79.5	0.2 (0.08–0.52)	0.01*	–	–
Median IQR (days)	20 (14–31.8)		15 (10.5–24.5)		0.98 (0.95–1)	0.08	–	–
**Use of drain**	16	7.1	5	11.4	1.67 (0.58–4.84)	0.34	–	–
Median IQR (days)	9 (4.8–11)		7 (6–8)		1.04 (0.96–1.14)	0.32	–	–
**Use of bladder catheter**	13	5.8	1	2.3	0.38 (0.05–2.98)	0.36	–	–
Median IQR (days)	3 (2–7)		4 (4–4)		0.92 (0.57–1.5)	0.74	–	–
**Classification of infection agent**								
GPB	257	66.4	35	50.7	0.52 (0.31–0.87)	0.01*	–	–
GNB	111	28.7	27	39.1	1.6 (0.94–2.72)	0.08	–	–
Fungus	19	4.9	7	10.2	2.19 (0.88–5.42)	0.09	–	–
**Susceptibility oftheagent**								
Sensible	59	15.2	10	14.6	0.94 (0.46–1.95)	0.87	–	–
Resistant	89	23.0	21	30.4	1.22 (0.73–2.06)	0.45	–	–
MDR	230	59.4	37	53.6	0.88 (0.52–1.47)	0.62	–	–
XDR	9	2.4	1	1.4	0.62 (0.08–4.95)	0.65	–	–
**Resistance mechanism oftheagent**								
ORS	207	53.5	30	43.5	0.67 (0.4–1.12)	0.13	–	–
ESBL	11	2.8	4	5.9	2.1 (0.65–6.81)	0.21	–	–
CR	1	0.3	1	1.5	5.68 (0.35–91.84)	0.22	–	–
**Site of infection**								
Bloodstream	250	64.6	59	85.5	3.23 (1.6–6.52)	0.01*	–	–
Eyes	98	25.3	6	8.8	0.28 (0.12–0.67)	0.01*	–	–
Genitourinary tract	31	8.1	2	2.9	0.34 (0.08–1.47)	0.15	–	–
Ascitic fluid	4	1.0	1	1.4	1.41 (0.16–12.79)	0.76	–	–
Cerebrospinal fluid	4	1.0	1	1.4	1.41 (0.16–12.79)	0.76	–	–

Among the discharge neonates, data were available on gestational age for 221, on type of delivery for 222 and on PROM for 197, whereas among the dead neonates, data were available on gestational age for 42, on type of delivery for 44 and on PROM for 34. During the period, there were 387 infections in the discharged group and 69 among those who died. All information after infection refers to the last episode.

Confidence intervals (CIs).

* *P*<0.05.

CI, confidential intervals; CR, carbapenem-resistant; ESBL, extended-spectrum beta-lactamase *Enterobacteriaceae*; GPB, Gram-positive bacteria; OR, odds ratio; PICC, peripherally inserted central catheter; PN, parenteral nutrition; UVC, umbilical venous catheter

The median length of stay in the NICU for infected newborns was significantly longer than that of the non-infected group (*P*=0.01), with a median of 42 days (IQR: 26–69), compared with 25 days (IQR: 13–47) in the non-infected group (Table 1). Among the infected, those who were discharged had a longer hospitalization (*P*=0.01), with a median of 46 days (IQR: 31–75), in comparison with 19 days (IQR: 10.8–26.8) for those who died (Table 2). The neonates in group 1 remained in the NICU for an even longer period (*P*=0.01), with a median of 55.5 days (IQR: 22–87.5), compared with 38 days (IQR: 27–58) for group 2.

### Epidemiological indicators

The infection incidence rate among VLBW newborns in the NICU was 40.1%, ranging from 34.4% in 2015 to 36.9% in 2022, reaching a peak of 52.1% in 2016. The mean incidence rate per 1,000 patient days was 10.2, varying from 12.3 in group 1 to 9.6 in group 2. In both groups, the highest infection densities occurred in 2016, with 24.5 and 16 per 1,000 patient days, respectively (Table 3).

**Table 3.** Epidemiological indicators of infections in very low weight neonates, below and above 750 g

Epidemiological indicator	Year of study								
	**2015**	**2016**	**2017**	**2018**	**2019**	**2020**	**2021**	**2022**	2015–**2022**
**Incidence of infection** (%)	34.4	52.1	38.4	41.4	41.1	41.9	40.0	36.9	40.1
<750 g	61.5	60.0	48.3	59.1	56.3	64.7	53.8	38.9	54.5
750–1,499 g	27.5	48.5	34.3	36.4	37.8	35.1	37.5	36.6	36.2
**Incidence of sepsis** (%)	18.8	39.6	31.3	30.3	32.2	31.1	31.8	28.8	30.3
<750 g	38.5	53.3	41.4	63.6	56.3	52.9	53.8	38.9	49.7
750–1,499 g	13.7	33.3	27.1	20.8	27.0	24.6	27.8	26.9	25.0
**Incidence of infection/1,000 patient days**	11.3	18.3	12.1	11.0	9.9	9.0	8.7	8.1	10.2
<750 g	19.0	24.5	15.7	13.7	10.4	10.4	8.2	7.5	12.3
750–1,499 g	9.2	16.0	10.6	10.1	9.7	8.4	8.8	8.3	9.6
**Mortality** (%)	15.6	27.1	26.3	20.2	15.6	10.8	14.1	13.5	17.6
<750 g	46.2	73.3	69.0	63.6	43.8	23.5	30.8	33.3	50.3
750–1,499 g	7.8	6.1	8.6	7.8	9.5	7.0	11.1	9.7	8.7
**Lethality** (%)	13.6	24.0	31.6	19.5	13.5	6.5	17.6	4.9	16.4
<750 g	25.0	55.6	57.1	38.5	33.3	9.1	14.3	14.3	33.3
750–1,499 g	7.1	6.3	16.7	10.7	7.1	5.0	18.5	2.9	9.4
**Infections and pathogens** (%)	6.3	8.1	16.7	15.6	14.0	9.9	13.6	15.8	100
<750 g	7.5	8.7	16.1	21.1	14.9	7.5	13.0	11.2	35.3
750–1,499 g	5.8	7.8	16.9	12.5	13.6	11.2	13.9	18.3	64.7
GPB	5.8	6.5	18.1	15.8	17.5	10.6	10.6	15.1	64.0
<750 g	6.2	4.1	21.6	25.8	20.6	9.3	7.2	5.2	33.2
750–1,499 g	5.6	7.7	16.4	10.8	15.9	11.3	12.3	20.0	66.8
GNB	6.5	13.0	10.9	15.2	8.7	9.4	19.6	16.7	30.3
<750 g	7.7	19.2	5.8	11.5	7.7	5.8	23.1	19.2	37.7
750–1,499 g	5.8	9.3	14.0	17.5	9.3	11.6	17.4	15.1	62.3
Fungus	11.6	0	30.8	15.4	3.8	3.8	15.4	19.2	5.7
<750 g	16.7	0	16.7	25.0	0	0	16.7	25.0	46.2
750–1,499 g	7.1	0	42.4	7.1	7.1	7.1	14.3	14.3	53.8

The incidence rate of sepsis was 30.3%, ranging from 18.8% in 2015 to 28.8% in 2022, reaching a peak of 39.6% in 2016. Similarly, the incidence of sepsis was higher in group 1 (49.7%) compared with group 2 (25.0%), mirroring the trend observed for HAIs (54.5% and 36.2%) (Table 3).

### Characterization of infections

**Fig. 1.**
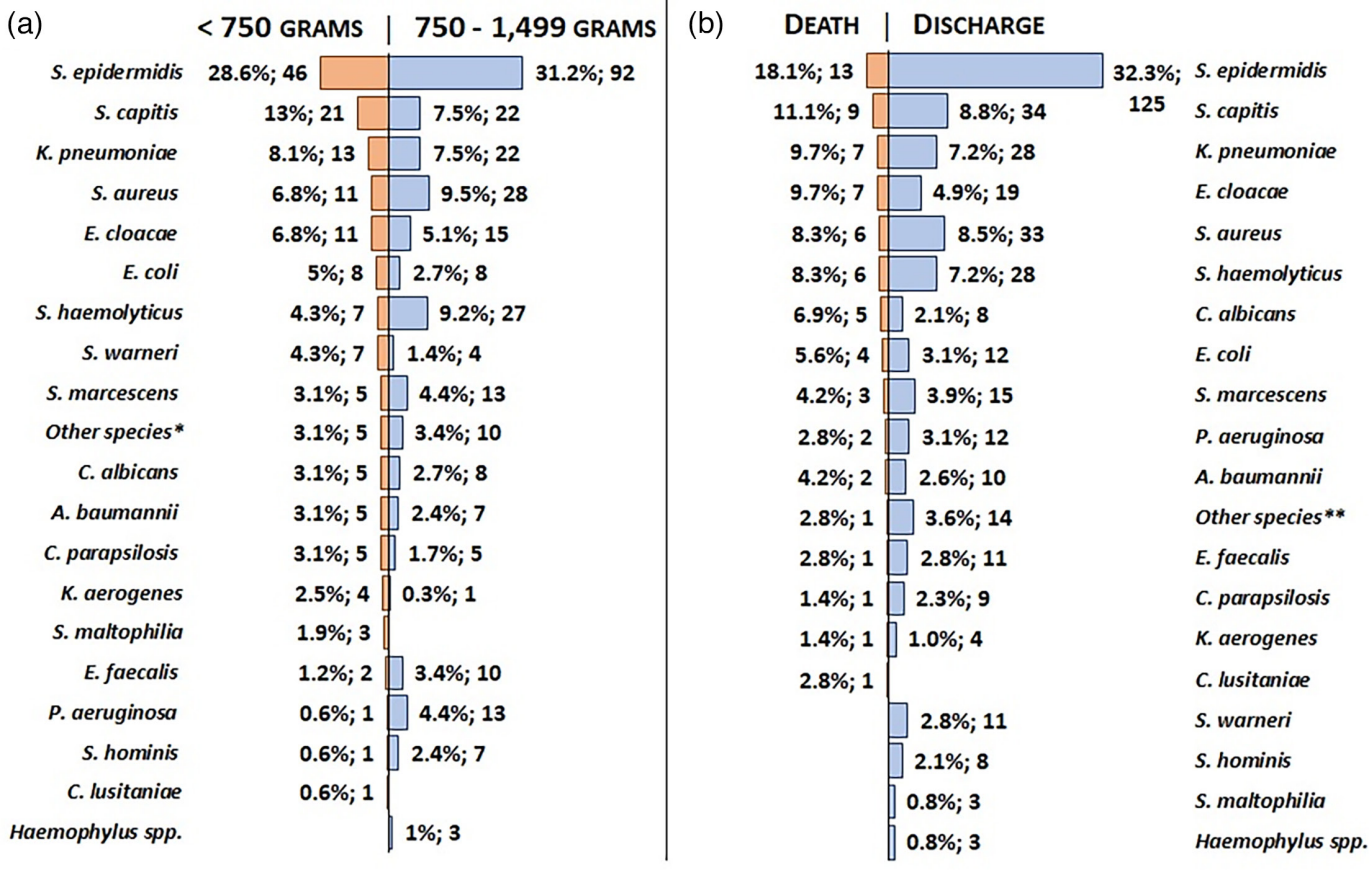
Etiological agents of infections in VLBW neonates below and above 750 g (a) and outcomes of those who died or were discharged (b).*<750 g: *Candida glabrata, Pantoea spp., Pseudomonas putida*, *Staphylococcus pseudointermedius and Streptococcus spp*. 750–1499 g: *Burkholderia cepacia, Cronobacter sakazakii, Klebsiella oxytoca, Listeria spp*.*, Sphingomonas paucimobilis, Staphylococcus carnosus, Staphylococcus intermedius, Staphylococcus lugdunensis, Streptococcus agalactiae* and *Trichosporon spp*.**Death: *P. putida.* Discharge: *B. cepacia, C. glabrata, C. sakazakii, K. oxytoca, Listeria spp*.*, Pantoea spp., S. paucimobilis, S. carnosus, S. intermedius, S. lugdunensis, S. pseudointermedius, S. agalactiae, Streptococcus spp*. and *Trichosporon spp*.

Out of 269 infected newborns, 103 exhibited multiple infections, including 27 polymicrobial, resulting in a total of 456 infections with 35 distinct species. Of the isolates, 292 (64%) were identified as Gram-positive bacteria (GPB), 138 (30.3%) as Gram-negative bacteria (GNB) and 26 (5.7%) as fungi. The predominant species were *Staphylococcus epidermidis* (138; 30.3%), *Staphylococcus capitis* (43; 9.4%), *Staphylococcus aureus* (39; 8.6%), *Klebsiella pneumoniae* (35; 7.7%) and *Staphylococcus haemolyticus* (34; 7.5%) (Fig. 1).

The incidence of infection caused by GNB (32.3%) and fungi (7.5%) was higher among newborns in group 1, whereas those in group 2 showed rates of 29.2% for GNB and 4.7% for fungi (Fig. 1a). A higher proportion of infections by GNB (39.1%) and fungi (10.1%) was observed among neonates who died compared with the discharged group, which had rates of 28.7% for GNB and 4.9% for fungi (Fig. 1b).

**Fig. 2.**
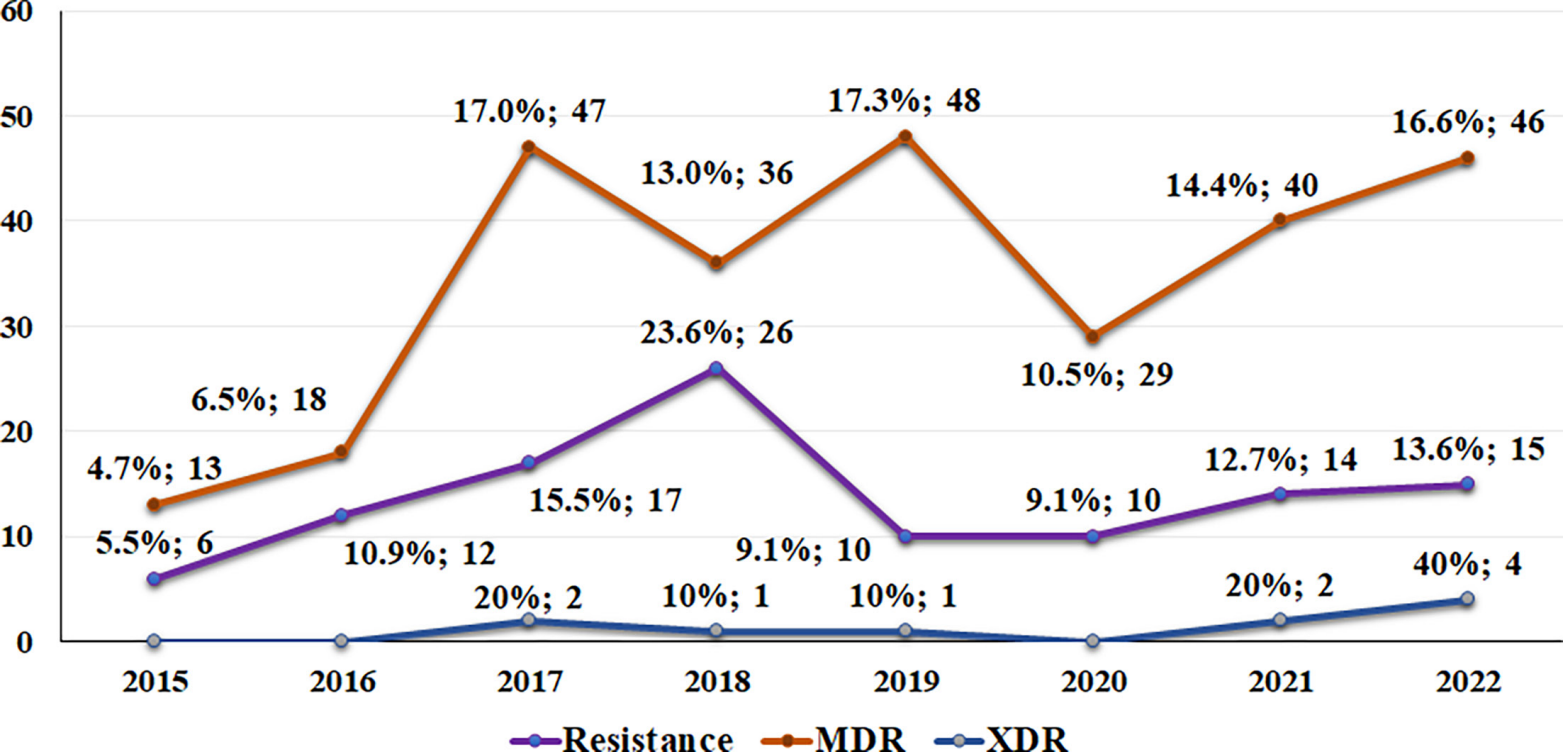
Annual distribution of antimicrobial resistance among aetiological agents of infections in VLBW neonates.

**Fig. 3.**
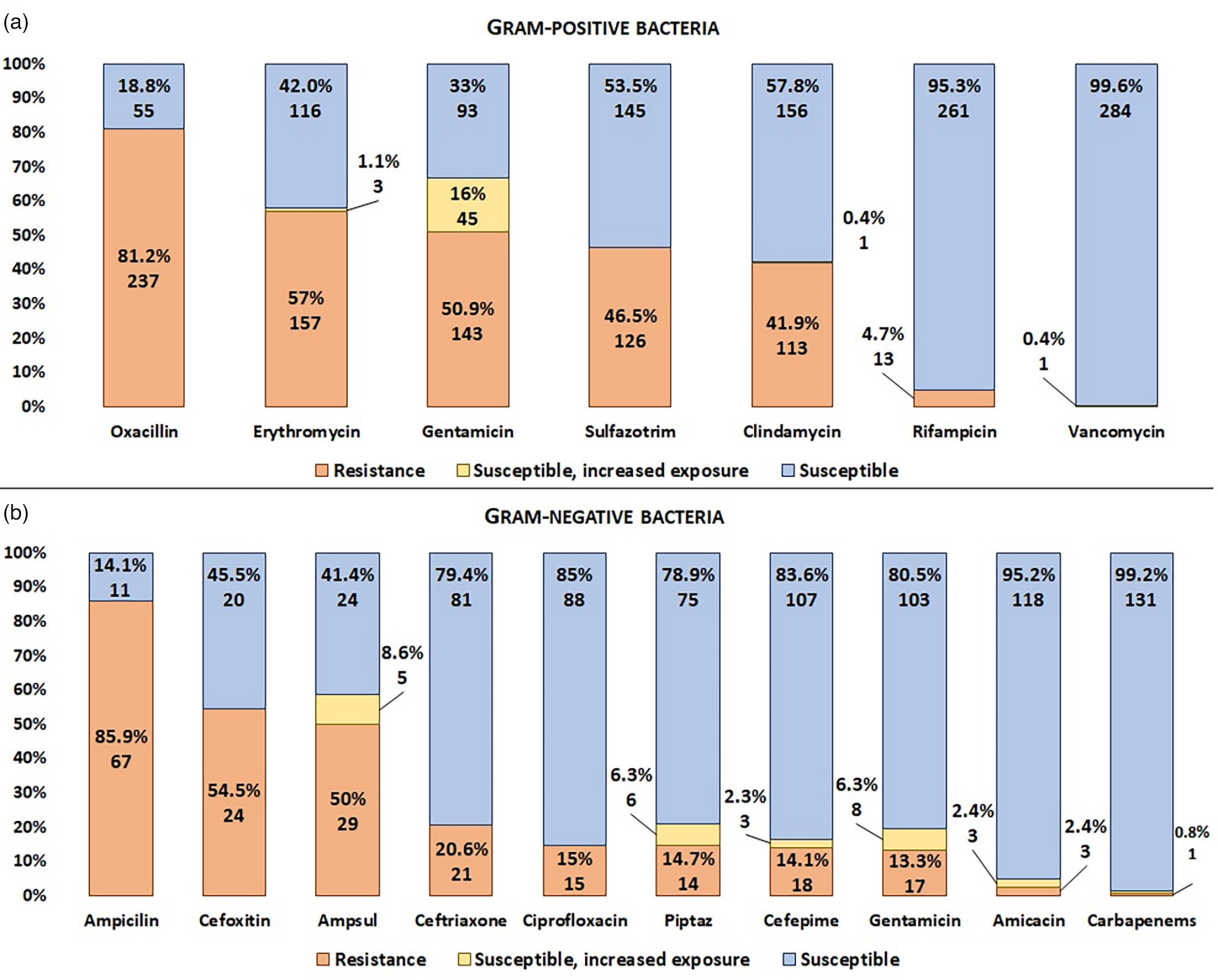
Antimicrobial resistance of the aetiological agents of infections in very low weight neonates. Ampsul: Ampicillin+sulbactam, Piptaz: Piperacillin+tazobactam.

Of the 456 isolates examined, 277 (60.7%) were MDR, with the majority (251; 90.6%) belonging to the GPB. After a peak in MDR cases in 2018 (23.6%), a temporary reduction was observed, followed by a notable increase between 2021 and 2022 (Fig. 2). Furthermore, 10 isolates were identified as XDR, with 70% of them being GPB. The XDR pathogen species included *S. haemolyticus* (6), *K. pneumoniae* (2), *S. epidermidis* (1) and *Enterobacter cloacae* (1). Further details on the antimicrobial resistance profile can be found in Fig. 3.

The infection sites were identified as the bloodstream (309; 67.8%), ocular secretion (104; 22.8%), genitourinary tract (33; 7.2%), ascitic fluid (5; 1.1%) and cerebrospinal fluid (5; 1.1%). Newborns in group 2 had a higher likelihood of developing bloodstream infections [*P*=0.05; odds ratio (OR)=1.14 (1.0–1.31)] and genitourinary tract infections [*P*=0.01; OR=2.27 (1.36–3.78)] compared with those in group 1. Conversely, the likelihood of developing conjunctivitis was lower in newborns in group 2 [*P*=0.01; OR=0.5 (0.32–0.78)].

### Risk factors for infections

In the univariate analysis, intrinsic risk factors for infection were identified, including birth weight less than 750 g [*P*<0.01; OR=2.11 (1.45–3.07)], extreme prematurity [*P*<0.01; OR=1.92 (1.39–2.66)] and congenital malformation [*P*<0.01; OR=1.85 (1.25–2.74)]. In the multivariate analysis, the primary predictor of infection was prior use of antifungals [*P*<0.01; OR=4.21 (1.85–9.58)], followed by duration of parenteral nutrition use [*P*<0.01; OR=1.06 (1.02–1.10)] and use of peripherally inserted central catheter (PICC) [*P*<0.01; OR=1.04 (1.01–1.08)] (Table 1).

### Lethality predictors and their indicators

The mortality rate was 17.6%, with a lethality rate of 16.4% (Table 3). The lethality rate was higher in group 1 (33.3%) than in group 2 (9.4%). The median birth weight was 980 g (IQR: 770–1,210) for those discharged from hospital and 668 g (IQR: 611.5–1,001.3) for those who died (Table 2). The interval between infection and death was 7 days (IQR: 2–15), statistically (*P*=0.01) longer in group 1 (8.5 days, IQR: 2–14.3) compared with group 2 (5 days, IQR: 2–30.5).

In the multivariate analysis, the primary extrinsic predictors of death were identified as treatment with fourth-generation cephalosporins [*P*<0.01; OR=5.89 (1.44–24.13)] and glycopeptides [*P*=0.02; OR=1.14 (1.02–1.27)]. In the univariate analysis, other significant predictors included intubation [*P*<0.01; OR=5.08 (1.52–17.02)] and bloodstream infection [*P*<0.01; OR=3.23 (1.60–6.52)] (Table 2).

**Fig. 4.**
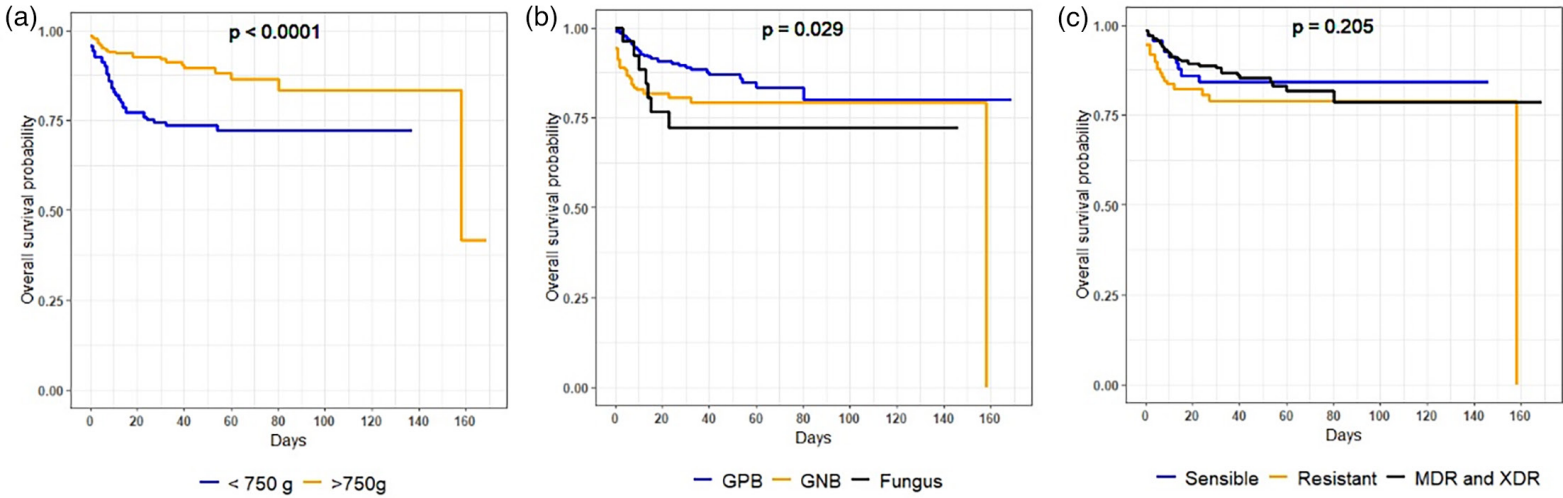
Distribution of infections in VLBW neonates relative to the Kaplan–Meier survival curve over time.

Especially during the initial 40-day period following birth, neonates in group 1 exhibited markedly inferior survival rates in comparison with those in group 2 (*P*<0.01). Furthermore, the survival rates were found to be lower in cases of infection caused by GNB or fungi compared with GPB infections, with statistically significant differences (*P*=0.02). However, the influence of antimicrobial resistance on survival did not attain statistical significance (*P*=0.20) (Fig. 4).

It is noteworthy that 75% (33/44) of the deaths in the infected group were attributed to sepsis. Among these neonates, 69.7% (23/33) were assigned to group 1. The 33 isolates that resulted in fatal sepsis were *Staphylococcus spp*. (10; 30.3%), *Candida spp*. (6; 18.2%), *Klebsiella spp*. (4; 12.1%), *Enterobacter spp*. (4; 12.1%), *Escherichia coli* (3; 9.1%), *Pseudomonas spp*. (3; 9.1%), *Acinetobacter spp*. (2; 6.1%) and *Serratia marcescens* (1; 3%).

## Discussion

In 2020, 19.8 million neonates worldwide were born with low birth weight (<2,500 g), a condition that is strongly associated with increased neonatal morbidity and mortality [18]. The present study, conducted over 8 years in a Brazilian public NICU, provides updated data on HAIs in VLBW newborns. This information is essential for the development of more effective preventive and therapeutic strategies aimed at reducing morbidity and mortality in NICUs.

Research on HAI in VLBW neonates is limited, with few studies focusing on this high-risk group and typically covering short analysis periods, unlike rare historical series such as the one presented in this article. Varied incidences of HAI have been observed in hospitals in China (22.4% between 2017 and 2019) [19] and South Africa (22.7% between 2016 and 2017) [6], as well as NICUs in Brazil (39% between 2008 and 2017) [20], the USA (29% between 2004 and 2010) [7] and Japan (5.3% between 2014 and 2015) [21].

The incidence of HAI in VLBW neonates in the NICU at HC-UFU was 40.1%, while the incidence of sepsis was 30.3%. This result was greater than the 23.7% late-onset sepsis (LOS) observed between 2006 and 2008 in NICUs of the Brazilian Neonatal Research Network [8] and the 25% sepsis rate between 2014 and 2015 within the same network [21]. However, the incidence of HAI and sepsis decreased from 2017 onwards, with the lowest values observed in 2022, at 36.9% and 28.8%, respectively.

The reduction in the incidence of infection over the course of this study suggests improvements in healthcare practice, driven by enhancement interventions, strengthening team collaboration and refinement of clinical procedures. The change in empirical antimicrobial choice, focusing on aminoglycosides and penicillins, also contributed to this reduction. These findings highlight the positive impact of multidisciplinary approaches in preventing HAIs in the NICU and support other studies conducted in the same unit [22,24].

In 2016, there was a peak in the incidence rate of HAIs and sepsis among VLBW infants in the NICU, reaching 52.1% and 39.6%, respectively. This increase may be related to the higher admission of critically ill neonates requiring broad-spectrum empirical antibiotic therapy, making them more susceptible to infection [25]. The extensive use of broad-spectrum antibiotics in the NICU in recent years to reduce LOS supports this possibility [26].

The fact that all infected newborns were premature emphasizes the vulnerability of VLBW preterm infants to infection. VLBW may result from both preterm birth and intrauterine growth restriction, or both [18]. This finding reinforces concerns about prematurity, especially in developing countries where the incidence of preterm birth is higher and access to obstetric and neonatal care is limited [27].

Prolonged use of venous catheters increases the risk of HAIs in VLBW infants [28], as demonstrated in this study, where PICC use was associated with an increased risk (OR=1.04) in the multivariate analysis. The predominance of GPB infections, especially coagulase-negative *Staphylococcus spp*. (CoNS), underlines the severity of these complications [29].

The predominance of GPB infections (64%) among VLBW infants reflects a trend observed in Brazilian NICUs [8,20, 25]. While low- and middle-income countries have a higher incidence of LOS caused by GNB, Brazilian NICUs show a prevalence of GPB, possibly due to hospital environmental conditions and prevention strategies such as hand hygiene [30].

There has been an increase in invasive fungal infections (IFIs) in newborns weighing less than 750 g between 2021 and 2022, which is associated with higher mortality. These infections are of concern in VLBW infants due to their developing immune systems. The symptoms of IFI can resemble those of bacterial infections, leading to inappropriate use of broad-spectrum antibiotics. This practice may worsen the clinical condition and increase complications and mortality rates in this population [10].

VLBW infants are at increased risk of IFIs, with incidence rates ranging from 1% to 7.5% and mortality rates that can reach up to 19.3% [31]. In this study, 5.7% of the isolates were fungi, with 96.2% being *Candida spp*. The mortality associated with IFIs was high, reaching 27.3%, compared with the overall mortality of 16.4%. These results underline the extreme severity of these infections in VLBW infants and emphasize the importance of appropriate treatment in the NICUs.

Antifungal prophylaxis is recommended for VLBW infants or <27 weeks of gestation with risk factors, or in NICUs with IFI rates ≥5% [32]. In the NICU at HC-UFU, the IFI rate is ~2% [24]; hence, antifungal prophylaxis is not administered. However, the use of antifungals as empirical treatment was observed in 19% of cases, resulting in a 4.2-fold increase in the risk of infection. It is believed that the low mortality was attributed to early empirical use of antifungals, which can reduce *Candida spp*.-related mortality by more than 90% [32].

This study, aligned with previous research, highlights the predominance of neonatal HAIs caused by CoNS [29]. Due to their high resistance to beta-lactams, with 81.2% resistance to oxacillin among GPB, vancomycin has emerged as a therapeutic option. Regarding GNB, treatments with aminoglycosides and carbapenems have shown efficacy [33]. Resistance to gentamicin was low (13.3%) among GNB. The emergence of carbapenem-resistant isolates underlines the need for continuous monitoring of antimicrobial resistance and adaptation of therapeutic strategies to ensure neonatal safety.

In the multivariate analysis, treatment with fourth-generation cephalosporins (OR=5.89) and glycopeptides (OR=1.14) emerged as predictors of mortality. These antibiotics are frequently used to treat MDR infections. Their use may reflect the severity of the infections faced, contributing to the worsening of clinical conditions and increasing the risk of death. Therefore, the need for such antibiotics may indicate severe and difficult-to-treat infections [34].

Of the 456 isolates in the study, 60.7% were MDR. Infections caused by MDR micro-organisms in neonates limit the effectiveness of first-line therapies, often requiring broad-spectrum antimicrobials like carbapenems or fourth-generation cephalosporins. This increases adverse effects and resistance, creating a cycle of fewer treatment options and worsening outcomes, such as delayed therapy, higher treatment failure and increased morbidity and mortality [35]. In low- and middle-income countries, limited access to effective antimicrobials exacerbates these issues, highlighting the need for strong antimicrobial stewardship and infection control to improve neonatal outcomes [36].

LBW newborns have a higher risk of mortality compared with those born at term [37]. Multivariate analyses have shown increased neonatal mortality rates in NICUs, ranging from 3.4 to 8.7 times in various locations such as Eritrea [38], Somalia [39], Ethiopia [40] and China [19]. In South America, mortality among LBW newborns reaches 26%, highlighting socioeconomic challenges in the region [27]. In this study, the mortality rate was 17.6%, exceeding the rates observed in NICUs in the USA (9%) [7], Brazil (16.1%) [20] and hospitals in South Africa (16%) [6].

In the NICU at HC-UFU, the overall mortality rate was 17.6%, with 16.4% among the infected group. This contrasts with results from NICUs in the USA, where mortality was higher among infected neonates (16% vs. 7%) [7]. In other Brazilian NICUs, mortality was higher in neonates with LOS (26.6% vs. 9.4%) [8]. The rigorous implementation of early detection protocols, prompt treatment and continuous monitoring of neonates in the unit analysed in this study may have contributed to the lower mortality observed.

Although infected neonates in this study have a lower mortality rate than the overall average, they showed significantly longer stays in the NICU, especially those with a birth weight below 750 g. This prolonged stay, supported by other studies [6], is attributed to their immunological fragility, increased susceptibility to HAIs, need for intensive care and increased risk of complications. Additionally, infected neonates often require prolonged and intensive treatment to mitigate associated complications [41].

The study highlights the critical vulnerability of neonates weighing less than 750 g, with 75% of deaths in the infected group due to sepsis, and 69.7% of these infants in the low-weight category. The average time from infection to death was only 7 days, and even shorter in the most vulnerable group (5 days). This group experienced higher rates of HAIs (54.5% vs. 36.2%) and sepsis (49.7% vs. 25%), along with increased mortality and longer NICU stays. The increase in IFIs between 2021 and 2022 further exacerbated mortality rates. Low birth weight (<750 g) was a significant risk factor for infection (*P*<0.01; OR=2.11), highlighting the need for targeted infection prevention and treatment strategies to improve outcomes in this high-risk group.

This study stands out for addressing HAIs in VLBW infants in a Brazilian NICU over an extensive and recent period (2015–2022). By comparing neonates weighing less than and more than 750 g, it provides insights into personalized management strategies. The analysis over 8 years revealed trends and the effectiveness of the interventions implemented, supporting the implementation of infection control measures in other similar NICUs and highlighting sustained success over time.

However, this long-term analysis faced variability due to the transition to electronic medical records starting from 2018, as well as structural and ancillary changes within the NICU. These factors were carefully considered to maintain the consistency of our findings. This study has inherent limitations due to its retrospective cohort nature, such as potential selection biases and incomplete information in medical records. Furthermore, as the study was conducted in a single NICU, the results may not be generalizable to other institutions or neonatal healthcare settings. Therefore, prospective and multicentre studies are needed to validate and extend these findings.

This study highlights the urgent need for improved infection control in NICUs, especially for VLBW neonates who are highly susceptible to HAIs and sepsis. The presence of MDR bacteria highlights the challenge of antimicrobial resistance, demanding stricter infection control and antimicrobial stewardship. The increase in GNB and fungal infections calls for innovative prevention and treatment strategies. High mortality rates further emphasize the need for comprehensive surveillance and rigorous hygiene protocols to ensure neonatal safety, urging continuous intervention and protocol adaptation to improve NICU outcomes.

## Abbreviations

CoNS: coagulase-negative *Staphylococcus* spp.
GNB: Gram-negative bacteria
GPB: Gram-positive bacteria
HAIs: healthcare-associated infections
HC-UFU: Hospital de Clínicas da Universidade Federal de Uberlândia
IFIs: invasive fungal infections
IQR: interquartile range
LOS: late-onset sepsis
MDR: multidrug-resistant
NICUs: neonatal intensive care units
PICC: peripherally inserted central catheter
PROMs: premature rupture of membranes
VLBW: very low birth weight
XDR: extensively drug-resistant
